# Revolutionising food advertising monitoring: a machine learning-based method for automated classification of food videos

**DOI:** 10.1017/S1368980023002446

**Published:** 2023-12

**Authors:** Michele Bittencourt Rodrigues, Victória Pedrazzoli Ferreira, Rafael Moreira Claro, Ana Paula Bortoletto Martins, Sandra Avila, Paula Martins Horta

**Affiliations:** 1 Nutrition Department, Federal University of Minas Gerais. Av. Alfredo Balena 190, 30130-100. Escola de Enfermagem, 3º andar, sala 312, Belo Horizonte, Minas Gerais, Brazil; 2 Institute of Computing, University of Campinas, Campinas, SP, Brazil; 3 Department of Nutrition, School of Public Health, University of São Paulo, Av. Dr. Arnaldo, 715 - Cerqueira César, São Paulo, SP, 01246-904, Brazil; 4 Center for Epidemiological Research in Nutrition and Health, Department of Nutrition, School of Public Health, University of São Paulo, Av. Dr. Arnaldo, 715 - Cerqueira César, São Paulo, SP, 01246-904, Brazil

**Keywords:** Food advertising, Monitoring, Ultra-processed foods, Artificial intelligence, Machine learning

## Abstract

**Objective:**

Food advertising is an important determinant of unhealthy eating. However, analysing a large number of advertisements (ads) to distinguish between food and non-food content is a challenging task. This study aims to develop a machine learning-based method to automatically identify and classify food and non-food ad videos.

**Design:**

Methodological study to develop an algorithm model that prioritises both accuracy and efficiency in monitoring and classifying advertising videos.

**Setting:**

From a collection of Brazilian television (TV) ads data, we created a database and split it into three sub-databases (i.e. training, validation and test) by extracting frames from ads. Subsequently, the training database was classified using the EfficientNet neural network. The best models and data-balancing strategies were investigated using the validation database. Finally, the test database was used to apply the best model and strategy, and results were verified with field experts.

**Participants:**

The study used 2124 recorded Brazilian TV programming hours from 2018 to 2020. It included 703 food ads and over 20 000 non-food ads, following the protocol developed by the INFORMAS network for monitoring food marketing on TV.

**Results:**

The results showed that the EfficientNet neural network associated with the balanced batches strategy achieved an overall accuracy of 90·5 % on the test database, which represents a reduction of 99·9 % of the time spent on identifying and classifying ads.

**Conclusions:**

The method studied represents a promising approach for differentiating food and non-food-related video within monitoring food marketing, which has significant practical implications for researchers, public health policymakers, and regulatory bodies.

Estimates from the World Health Organization (WHO) show that, in 2016, more than 1·9 billion adults were overweight, with more than 650 million obese^(1)^. Obesity is a chronic disease, in addition to being one of the main risk factors for several other non-communicable diseases (NCDs), such as cardiovascular diseases (CVD), hypertension, stroke, certain types of cancer and diabetes^(2,3)^, the leading cause of death worldwide^(4)^. Unhealthy diets are a major contributor to the epidemic of overweight and obesity^(5)^ and thus have a central role in this scenario.

Unhealthy diets are promoted through food advertisements (ads)^(2)^, centred on products high in saturated fats, sodium, added sugars, and low in protective nutrients, such as fibres, vitamins, and minerals^(2,6,7)^. The advertising message has a high convincing power through persuasive marketing strategies such as ad repetition, product demonstration, peer popularity appeal, celebrity endorsement, and awards^(8,9)^. Consequently, exposure to food advertising can impact consumer behaviour, stimulate the desire for the product and retain consumer loyalty^(10–13)^. These effects can be noted in different groups of individuals, although more emphasis has been put on children, an audience that lacks the cognitive skills to understand the persuasive intent of ads^(12,14,15)^. Therefore, restricting food advertising has been a global priority for health organisations that encourage civil society organisations, academic researchers, and governments to monitor and address the problem^(16–18)^.

In a publication aimed at guiding governments on the harmful impact of food marketing, WHO and the United Nations Children’s Fund (UNICEF) emphasise the crucial role of monitoring in policy-making related to the impact of food marketing on children^(19)^. Monitoring provides data for assessing policy effectiveness and quantifying the extent of exposure to unhealthy marketing^(19)^. Policymakers utilise this data to refine existing policies and identify regulatory shortcomings^(19)^. Additionally, monitoring aids in the detection of non-compliance and emerging marketing trends, ensuring policy relevance^(19)^. Furthermore, it fosters public awareness and enhances accountability by disseminating information about the impact of marketing and mobilising support for stricter regulations and responsible industry practices^(19)^.

Currently, ads are disseminated through various media channels, including television (TV), radio and the Internet. Effectively monitoring these ads presents several challenges, including the analysis of extensive volumes of media content, resulting in sluggish data processing, high costs for data collection, slow study execution and the potential for errors arising from manual work.

One of the primary difficulties in manual monitoring is identifying and differentiating food advertising from non-food advertising due to the high volume of information that must be processed manually, which demands a large and well-trained team. Consequently, the quantity and quality of evidence produced may be insufficient, impeding its translation into effective public policies. Experts emphasise the potential benefits of employing artificial intelligence (AI) in evaluating food marketing^(20,21)^. These advantages include scalability, reproducibility, consistency and the capacity to capture marketing content embedded within audio, text, still images, videos and even immersive experiences such as virtual reality^(20)^. Moreover, researchers contend that AI is indispensable for the comprehensive and systematic examination and monitoring of food marketing practices. Consequently, the proposal for automated monitoring holds promises in supporting the implementation of comprehensive policies aimed at reducing children’s exposure to unhealthy food marketing.

Researchers in Canada and Australia are developing AI systems to monitor food marketing targeted at children^(22–24)^. The Canadian AI system focuses on monitoring digital platforms^(22,23)^, while the Australian machine learning (ML) algorithms include both digital and non-digital contexts^(24)^. Although these studies are currently in the protocol phase, the outcomes of the AI-based system for automatic monitoring have not yet been published^(22–24)^. Therefore, the primary aim of this study was to develop a ML-based method that could automatically identify and classify food and non-food ad videos by investigating deep neural networks.

## Methods

### Design and database

This is a methodological study that followed the Food Promotion Module: Food Marketing – Television protocol^(25)^, which has been developed by the INFORMAS network (International Food and Obesity/Noncommunicable Diseases Research, Monitoring, and Support Network)^(26)^ for collecting TV ads. The primary objective of this protocol is to establish a standardised framework for collecting television data and systematic programming to evaluate the influence and prevalence of unhealthy food promotion on TV.

The database consisted of recorded TV broadcasts of three free-to-air channels in April 2018, May 2019 and June 2020 in Brazil. In addition, two pay-for-view channels were monitored in May 2019, September 2019 and June 2020. The time periods were defined following the recommendations of the INFORMAS protocol^(25)^. For each round of data collection, the TV channel programming was recorded in digital format, by a company specialising in providing a clipping service, for eight non-consecutive days, randomly drawn, four of which were weekend days (Saturday or Sunday) and 4 weekdays (between Monday and Friday). The recording covered the entire programming of the channels from 6 am to 12 am, totalling 18 h a day for each channel and 2124 h in the 3 years of the study (due to a recording failure, 2 d (36 h) of a pay-for-view channel in June 2020 were lost).

### Data coding

After the recordings were completed, the content was audited in five steps:

*(i)* audit carried out by the company responsible for clipping service: the company identified the total number of ads and coded the ads for food, beverages, and food retail outlets. Ads related to food and beverages were individually identified.

*(ii)* audit performed by trained researchers: two trained researchers independently audited the recording. Both researchers did a general check on the number and timing of videos recorded. With this audit, it was possible to identify the absence of some periods or even duplication of videos.

*(iii)* data tabulation: five pairs of ten trained volunteers independently performed the data tabulation. Subsequently, the obtained results underwent a thorough verification process to identify and eliminate any potential divergences between the researchers who carried out the data tabulation. For all ads, the following information was extracted: the channel name; channel type (i.e. free-to-air or pay-for-view); date of recording (classified as a day of the week or weekend); programme name; advertising time (start and end); and type of advertising: *(a)* food-related ads; *(b)* food or drink company or brand (no retailer) without food or drink product; *(c)* food or drink retailer (supermarket or convenience store) with/without food or drink product; *(d)* food or drink retailer (restaurant or takeaway or fast food) with/without food or drink product; and *(e)* non-food or drink product(s). All ads were tabulated in an electronic questionnaire built in *Epi InfoTM*, version *7.2.2.6*.

*(iv)* final audit: following the data tabulation process, a comprehensive audit was conducted by two independent researchers to identify any divergences among the pairs involved in the tabulation. The inter-coder reliability rate ranged from 90·4 % to 96 %, indicating a high level of agreement between the researchers. This meticulous examination allowed for the identification of additional food and beverage ads that were not initially detected in the initial audit.

*(v)* database creation: after conducting the final audit, we created a database that contains information on all ads, including both food and non-food ads. Additionally, we generated a spreadsheet of highly detailed notes of all pertinent data on these ads, which proved indispensable for training the algorithm conducting this study.

It is worth noting that these five manual steps are necessary to organise the data for the subsequent stages of algorithm development.

### Data preprocessing

With the database created, and all ad annotations completed and audited, the subsequent step involved data preparation and preprocessing. Following advertising monitoring guidelines, only food ads were individually separated into distinct videos through manual processing, while non-food ads were interspersed within daily programming into several 10-min videos, necessitating automating the process of cutting these videos. To optimise this process, a Python code utilising ffmpeg^(27)^, a versatile cross-platform solution for recording, converting, and clipping audio and video streams, was developed to cut these videos from the comprehensive channel schedule based on previously annotated timestamps.

Once this step was implemented, we observed a significant presence of duplicate non-food ads within the recently extracted videos, accounting for approximately 80% of the collected videos. Consequently, these duplicates were removed from the database.

We emphasise that this is the first database of this type, and we make it publicly available. For this, we will follow the recommendations made in the article Datasheets for Datasets^(28)^.

### Data splits

**Fig. 1:**
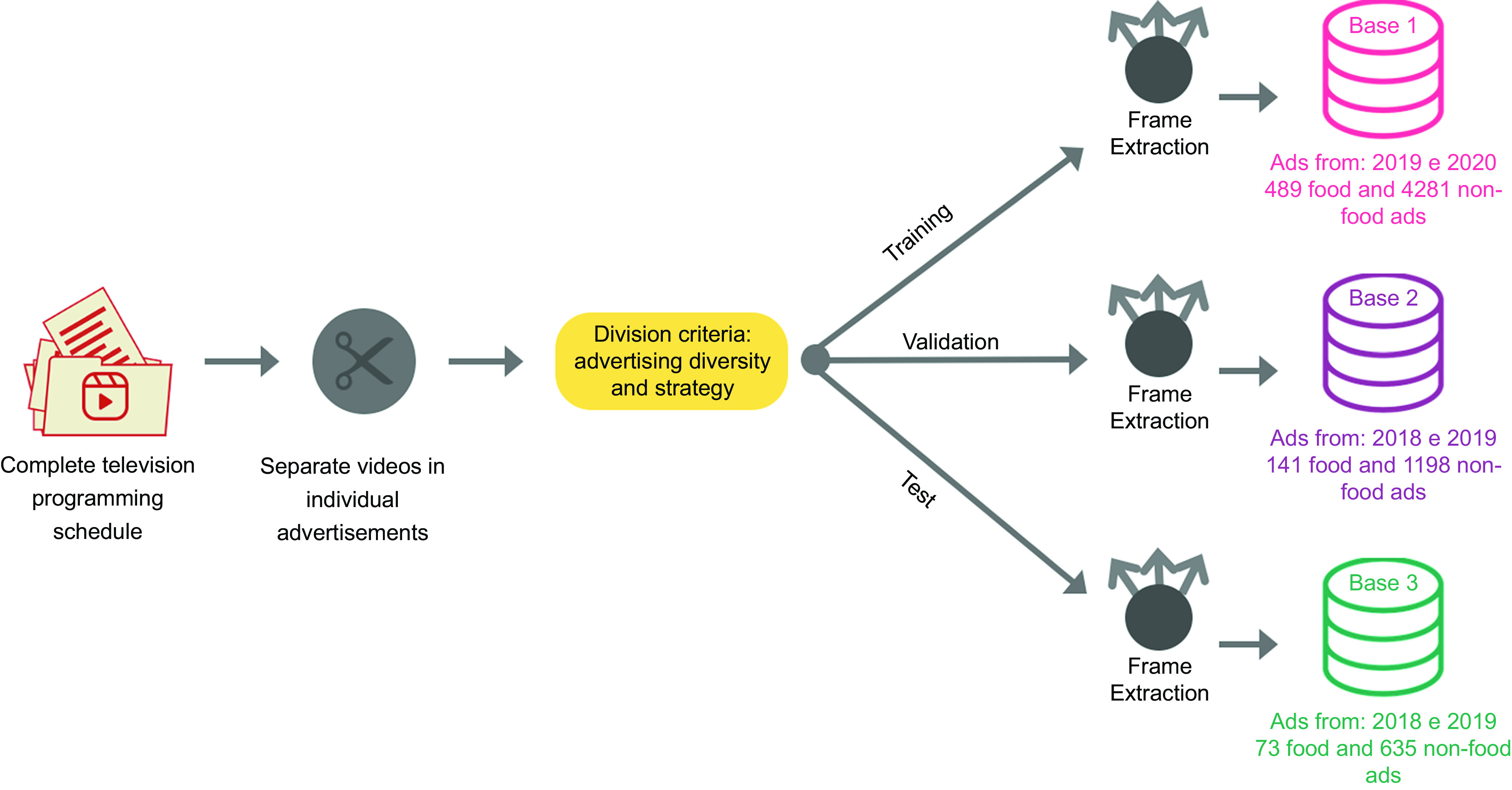
Synthesis of the methodology that comprises the database preprocessing and split stage

After data preprocessing, we split the database to train the ML model. We manually split the ‘initial database’ into three databases, as shown in Fig. 1: Base 1 for algorithm training, Base 2 for result validation and Base 3 for final model testing. We chose a manual and meticulous division over a random one because a random division might not consider certain important criteria for the model’s learning process. These criteria include the representation of all companies and types of advertisements, with a particular emphasis on food ads, across all three datasets. As a result, the division was carried out as follows:

*(i)* for Base 1, we included all videos from 2020 and videos collected in the second half of 2019, of which: 489 food videos and 4281 non-food videos, totalling: 4770 videos.

*(ii)* for Base 2, we included the videos from the first half of 2019 and part of the videos from 2018, of which: 141 food videos and 1198 non-food videos, totalling: 1339 videos.

*(iii)* for Base 3, we included only videos from 2018, except for a few cases of children’s channels, which were collected from 2019, of which: 73 food videos and 635 non-food videos, totalling: 708 videos.

This division was carried out considering the proportion of 75 % for training, 15 % for validation and 10 % for testing^(29)^. In ML, data division into subsets is vital to avoid overfitting, a common problem where the model becomes too specialised in the training data, so it loses its ability to generalise to new, unseen data^(30)^.

We divided the data in a way that allocated most of it for training the model while ensuring that both Base 2 and Base 3 still represent the entire initial database. Also, we applied specific strategies during the model training process due to the imbalanced data, where non-food ads were the majority. These strategies will be discussed later.

### Machine learning-based method

A ML-based method refers to an approach or technique used in the field of AI and computer science to solve problems or make predictions by leveraging ML algorithms. These algorithms are computational procedures or mathematical models used in the field of ML to enable computers and systems to learn and make decisions or predictions from data without being explicitly programmed.

For this study, we use the extracted frames from Base 1 (training data) and apply techniques such as data augmentation, balanced batches or class weights to mitigate data imbalance. Then, the frames go through the selected deep neural network (the EfficientNet) to generate a classification for each frame. After that, a fusion process through an average pooling method is used to get a single classification for the video by combining the ratings of each frame from the same advertisement. These processes result in a series of different trained networks (models). We determined the best strategies by validating the videos separated in Base 2 (validation data). This step indicates that some adaptations may be necessary during the training stage. Once no further changes are necessary, we select the best model obtained so far to be tested on Base 3 (test data), leading to results that can be verified and analysed by human experts in the field.

### Deep neural network: EfficientNet

A deep neural network is a computer system that learns and makes decisions by processing large amounts of data. It is used for tasks such as recognising faces in photos and objects and understanding language. Image recognition is a classic computational classification problem, and convolutional neural networks, specifically EfficientNets^(31)^, are the state of the art for this issue. The model’s effectiveness is highly dependent on the base architecture of the network. EfficientNet offers eight versions of this base architecture (EfficientNet-B0 to EfficientNet-B7). For example, EfficientNet-B0 has 5·3 million parameters, while B7 has 66 million. For context, consider teaching a child to identify fruits in images. EfficientNet-B0 is akin to teaching them to recognise common fruits, while B7 enables recognition of a wide variety, including exotic ones. Additionally, EfficientNet-B0 achieves 77·3 % top-1 accuracy on ImageNet, while EfficientNet-B7 reaches 84·3 %. In our study, we employ the best-accuracy version, EfficientNet-B7, which has been pre-trained on a substantial dataset of images (ImageNet dataset). This pre-training equipped the model to recognise a wide array of foods and objects. Subsequently, we fine-tune it for our specific task of identifying food and non-food ads, ensuring it provides the most precise results for our research.

### Approach to classification

Using Base 1, we propose classifying images and videos of food ads. As input to the neural network, for food ads videos, we used a frame sampling rate of one frame per second (1 fps), while for non-food ads videos, we selected the frame sampling rate as follows, where *T* is the duration of the video in seconds:

- 1/2 fps, if *T* < 10
- 1/4 fps, if 10 < *T* ≤ 25
- 1/6 fps, if 25 < *T* ≤ 35
- *T*/15 fps, if 35 < *T* ≤ 60
- *T*/30 fps if 60 < *T* ≤ 120
- *T*/45 fps, if *T* > 120

We opted for different frame sampling rates to mitigate the observed data imbalance during previous tests. All frames were standardised and centred according to the metric compatible with the input size of the chosen model: for EfficientNet-B7, the resolution varies from 224 × 224 to 600 × 600 pixels.

Each frame extracted from the ads was classified independently. To determine the video’s category (food or non-food ads), we employed a decision-making approach. We used simple and weighted average strategies to combine the video frame predictions into a single final prediction per video. However, we realised that using different frame sampling rates was not enough to overcome the data imbalance. To overcome this problem, we applied three strategies – data augmentation, balanced batches and class weights – to obtain the best result.

### Data augmentation

**Fig. 2:**
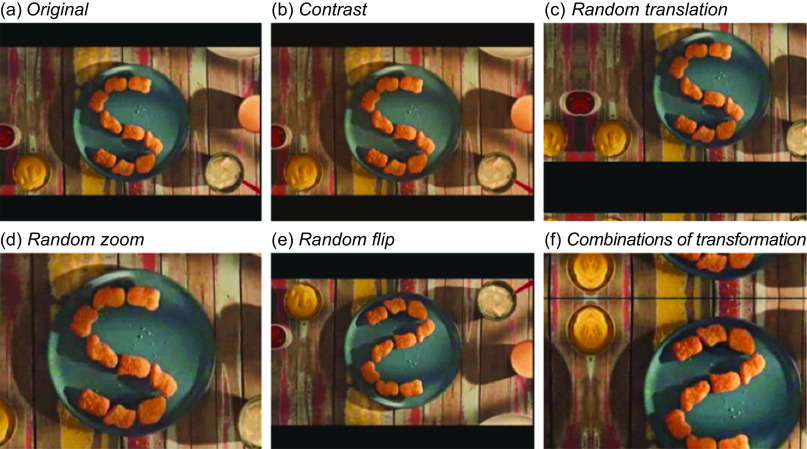
Examples of data augmentation transformations performed in the food advertising frame

Data augmentation refers to a set of techniques used in ML to artificially increase the size of training data by applying transformations to the original data. We apply the following transformations for the frames extracted from the food advertising videos: rotation, translation, zoom, adding or removing contrast, and rotations in the vertical and/or horizontal axis (Fig. 2). For that, we use the Keras library^(32)^, which allows the application of more than one transformation per image. At the end of this process, the class imbalance is attenuated by inserting new randomly generated data for the underrepresented class (food ads).

### Balanced batches

Balanced batching is a technique employed in the training of ML models with the objective of achieving a balanced distribution of classes within the training data. In ML, a batch represents a set of input data processed during training. In balanced batches, the number of samples from each class in the dataset is approximately equal in each batch. Balancing the batches exposes the ML model to diverse examples from all classes in each training iteration, leading to better generalisation performance and avoiding overfitting to any specific class.

In the context of this study, batching is a valuable approach applied to identify the presence of either food or non-food items in ads, particularly when dealing with a substantial volume of ad content. For instance, ads can be categorised into batches based on the type of products or services being promoted. For example, one batch may include ads related to fast-food restaurants, while another may comprise ads featuring fitness equipment.

We use the imbalanced-learn^(33)^, Python library, which creates a balanced data generator, repeating random samples from the database if there are not enough images to balance the classes within the batch in question. For instance, if there are 100 images of food items (the minority class) and 500 images of non-food items (the majority class), the goal is to construct a batch containing fifty images with balanced classes. The generator might randomly select fifty food item images and fifty non-food item images, with some food item images repeated as needed to attain the desired balance.

This approach ensures that the ML model is exposed to an equal number of examples from each class during training. As a result, the model’s capacity to accurately recognise and classify both classes is improved, even when dealing with highly imbalanced datasets.

### Class weights

Class weights are a valuable technique employed in ML classification tasks to address the issue of unbalanced datasets. This approach involves assigning different weights to various classes during the training of a model. The idea is to assign higher weights to the minority class (food ads) and lower weights to the majority class (non-food ads); thus, the model pays more attention to the minority class and learns to predict it more accurately. The class weights are typically calculated as the inverse of the class frequency.

We use the Scikit-learn^(34)^ Python library, which contains a function that estimates class weights for imbalanced databases.

We highlight the potential of utilising all three techniques in conjunction.

### Model training

The primary objective of this step is to instruct the ML model in distinguishing between food and non-food ads. In our study, we conducted a rigorous process of model training within the framework of Base 1 to determine which technique or combination of techniques provides the best results. The primary aim of this step is to teach the ML model to differentiate between food and non-food advertisements. In total, we performed eight experiments, seven including all possible combinations of the previously discussed techniques and one without applying any. To ensure robustness and account for variability in the outcomes, each experiment was replicated five times. This comprehensive approach resulted in the training of a total of forty distinct models. The replication of experiments allowed us to assess the consistency and reliability of the results, thereby enhancing the validity of our findings.

### Model validation

The validation set plays a crucial role in refining and optimising the model during the training process. It serves the dual purpose of selecting the most appropriate hyperparameters and assessing the model’s performance on unseen data. This intermediary step bridges the gap between training and testing, helping to prevent overfitting (where the model performs well on training data but poorly on new data).

We validate all the models in Base 2 to certify their predictions. For each of the forty models, we explored four methods to merge the frame rating into a video rating. This involved varying the weight assigned to the frames classified as part of our interest class (food ads) in the following weight values: 1, 2, 3 and 4, resulting in 160 different approaches. Also, we seek to find sources of predictive errors in the models, which will be discussed later. The metrics used to evaluate the models are balanced accuracy and confusion matrix.

### Model testing

Finally, we tested in Base 3 only the best-performing model with the best merging approach between the 160 possibilities. This step aims to evaluate its performance on data that the model has not yet had contact with, to get a more realistic idea of how this model would behave outside a controlled environment, classifying new ads. We also seek to find the sources of predictive errors in the model. The metrics used to evaluate the models are balanced accuracy and confusion matrix.

### Statistical analysis and analysis of algorithmic results

The main results of the trained models were portrayed by the video confusion matrices of the following sets. A confusion matrix is a table used to evaluate the performance of a classification model. Typically, it is used in ML and statistics to measure the overall accuracy of a model’s predictions. The matrix compares the predicted output of a model to the actual output and categorises the results into four groups: true positive (TP), true negative (TN), false positive (FP) and false negative (FN).

The TP prediction represents when the model correctly predicted the positive class (i.e. food ads), and the number of TN predictions represents when the model correctly predicted the negative class (i.e. non-food ads). Also, the FP prediction represents when the model predicted a positive class, but it was negative, and the FN prediction represents when the model predicted a negative class, but it was positive. Thus, the percentage of the number of FP, FN, TP and TN were portrayed in matrices.

To assess the model’s accuracy in predicting each video, we utilised the confidence level output provided by the algorithm. The confidence level indicates the model’s certainty or confidence in its classification decision, reflecting its belief in the accuracy of the prediction. Consequently, when categorising a video as either food or non-food, the model provided a confidence level alongside the predicted class label. This confidence level was expressed as a percentage, ranging from 0 % to 100 %.

To establish a criterion for the reliability of the model’s predictions, specialists in food advertising monitoring reviewed and analysed all the ads that were misclassified by the algorithm. We also adopted a threshold (empirically determined) of 65 % wherein the expert manually reviewed a video with a confidence lower than this rate (from 50 % to 65 %). By incorporating the review of videos correctly classified, but with a lower confidence level, we aimed to enhance the interpretability and reliability of the model’s predictions, enabling a more nuanced assessment of its performance to ensure the quality of the classification, specifically for food ads.

Both steps were done to identify the reasons that may have contributed to the incorrect classification.

## Results

We report the result of the best model combined with the best classification merging approach. Each table represents the execution of this specific experiment on different bases. Tables 1, 2 and 3 show the confusion matrix of food/non-food classification using EfficientNet-B7 associated with the technique of balanced batches with merging weight 2 for the classification of more than one frame per video for Base 1, 2 and 3, respectively.

**Table 1:** Classification confusion matrix for each class in the training database using EfficientNet-B7 for classification of more than one frame per video associated with the balanced batches technique with merging weight 2

Training at base 1	Food	Non-food
Food	100% (498/498)	0% (0/498)
Non-food	0·4% (15/4281)	99·6% (4266/4281)

**Table 2:** Classification confusion matrix for each class in the validating database using EfficientNet-B7 for classification of more than one frame per video associated with the balanced batches technique with merging weight 2

Validating at base 2	Food	Non-food
Food	90·1% (127/141)	9·9% (14/141)
Non-food	9·6% (115/1198)	90·4% (1083/1198)

**Table 3:** Classification confusion matrix for each class in the testing database using EfficientNet-B7 for classification of more than one frame per video associated with the balanced batches technique with merging weight 2

Testing at base 3	Food	Non-food
Food	90·4% (66/73)	9·6% (7/73)
Non-food	9·3% (59/635)	90·7% (576/635)

When evaluating the algorithm’s performance, we compared the overall accuracy of validating at Base 2 and testing at Base 3, which were 90·2 % and 90·5 %, respectively. The similarity between these results suggests that the algorithm performs consistently and effectively. Most promising, the slight differences observed between the results obtained from Base 2 and Base 3 highlight the importance of our careful data division approach. This approach ensured that the videos from the same round of data collection were not grouped in the same database, thus avoiding a negative impact on the model’s performance when tested on new data. Additionally, to optimise the model’s hyperparameters, the algorithm was validated multiple times on Base 2, which may lead to over-optimistic results. Nevertheless, the algorithm’s effectiveness was demonstrated by its single execution in Base 3, indicating its accuracy, robustness and reliability, thus enhancing its effectiveness in new data. To carry out the training, validation and testing step, we utilised two distinct machine configurations: *(i)* a GPU RTX 2080 with 11 GB of memory and *(ii)* a Titan Xp with 12 GB of memory. Notably, during the testing step, the algorithm consistently delivered results within a 20-min time frame, regardless of the machine’s specifications.

Upon analysis, specific patterns have been identified that contribute to the misclassification of ads by our algorithm across all three databases. Regarding non-food ads classified as food ads, we identified two main reasons for this misclassification. First, some non-food companies featured food or scenes of people eating or drinking in their ads. Second, some non-food ads shared similar characteristics with food ads, such as the use of colours (e.g. red and yellow) and the prices of products being advertised, like those featured in supermarket ads for food.

On the other hand, we also analysed food ads that were classified as non-food ads and found several reasons behind this misclassification. First, shorter ads lasting a maximum of 5 s featured fewer frames of food, making it difficult for the algorithm to classify them. Second, some food company ads did not feature any food or scenes of people eating or drinking, like McDonald’s ad for a contest for children to enter the field during the 2018 World Cup with the soccer player Neymar in a match for the Brazilian team. Third, some beverage ads only showed the liquid being poured into a glass, without focusing on the packaging of the product. Lastly, ads for food supplements only showed a person ingesting a capsule at a particular time, which was insufficient for the algorithm to classify them as food ads.

## Discussion

From the analysis of 2124 h of recording of Brazilian TV programming, using the Food Promotion Module: Food Marketing – Television protocol^(25)^, we created an initial database containing 703 different food advertising videos and more than 20 000 non-food advertising videos. Using this initial database, the study assessed the effectiveness of an ML model based on EfficientNet combined with strategies to alleviate the data imbalance in identifying and ranking food/non-food video ads. This identification and classification represent a major step in systematically monitoring this type of ad. Our results showed that the algorithm achieved an overall accuracy of 90·5 % in the test database, representing a great advance towards adopting an automatic monitoring approach.

The relevance of our study’s approach to automating the monitoring process of food marketing can address the challenges posed by collecting and analysing large volumes of data. Traditionally, studies have been limited to small samples due to the resource-intensive nature of the data collection and analysis process^(21)^. This limitation makes it difficult to monitor ads systematically across different media, time periods and population groups, hindering the ability to compare findings across countries. An automated approach could simplify the process, reduce the time and resources required for monitoring, and facilitate comprehensive monitoring of food marketing.

For example, if a research team chooses to record 18 h of TV programming during 8 d of three free-to-air and two pay-for-view channels, as recommended in the Food Promotion Module: Food Marketing – Television protocol^(25)^, a total of 720 h of programming must be recorded. The first task of this team is to be trained to identify and classify all instances of food and non-food ads and to watch the whole recorded content to separate these types of ads. Based on our experience, at least ten researchers are necessary to carry out this step within 2 weeks. Assuming that 1 d of programming requires approximately 10 h of work, the researchers would spend approximately 800 h only on the first stage of monitoring to identify a mean of 1460 unique ads. Using our test database, which contained 708 unique ads, we classified food and non-food ads in less than 20 min. Given this result, it would require approximately 42 min to analyse the sample of 1460 unique ads. Therefore, using the algorithm reduces 99·9 % of the time spent on the task, representing a significant financial resources savings achievement.

However, the method did not work with the same accuracy for all ads. Analysis of our test results revealed that the main classification error occurred in short ads (≤5 s) and those with less prominent food and/or beverage features. Interestingly, these errors were also common in the manual collection, where researchers occasionally overlooked these brief ads with minimal food scenes, leading to the omission of notes. However, in the manual collection, with the data double-entered, these errors were captured during the audit. By adopting automatic monitoring, we could verify and address these issues through an analysis of the confidence levels provided by the algorithm for each video classification. In addition, these errors primarily occur in ads with lower persuasive impact, and such errors are to be expected. Advertisements with lower persuasive impact refer to promotional materials or campaigns that have a reduced ability to influence or persuade their target audience. These advertisements may fail to effectively communicate the intended message, generate desired emotional responses or motivate viewers to take the desired action, such as making a purchase or changing their behaviour. For example, in our study, these are short ads that do not exploit emotional appeals (e.g. ads that aim to tap into the viewers’ emotions, evoking feelings such as joy, excitement or empathy) or visual effects (e.g. presence of food, cartoon or company-owned characters, celebrities, esthetically pleasing designs, the strategic use of colour, among others). However, since our database is still relatively small, we can assume that in the future, the algorithm will probably be able to improve its performance through the capacity of a larger volume of information.

To our knowledge, this is the first study to present results from automatic monitoring of food videos, highlighting the novelty of our study. In Canada, studies focus on automatic monitoring advertising on digital media^(22,23)^, proposing various steps for this approach, such as data collection, information extraction, marketing characteristics identification and nutritional information extraction^(22,23)^. The aim is to develop an AI-based system to monitor the marketing of unhealthy foods/brands to children on websites, social media and mobile gaming apps^(22,23)^, based on the CLICK monitoring structure proposed by WHO European Office for the Prevention and Control of Noncommunicable Diseases^(18)^. In Australia, researchers are also developing ML algorithms integrated into an image recognition system. This system autonomously detects and categorises detrimental food, alcohol, and tobacco advertising, aiming to monitor children’s exposure and engagement with such advertisements in both digital and non-digital contexts^(24)^. Despite the promising nature of these interdisciplinary studies, they are currently in the protocol phase, and the results of the AI-based monitoring system have not been published yet^(22–24)^.

Additionally, Palmer et al. developed a deep learning workflow to automatically extract and classify outdoor advertising promoting unhealthy products^(35)^. Through a comprehensive dataset comprising 25 349 georeferenced images collected through cycling expeditions equipped with GoPro cameras, the researchers successfully applied automated techniques to identify and classify a total of 10 106 ads. Among these, 1335 pertained to food, and 217 to alcohol, while the remaining 8554 encompassed various other products. This study provides a compelling demonstration of the efficacy of an automated approach in accurately classifying street view images to discern and categorise unhealthy ads. Consequently, this technique offers a valuable means to identify geographic regions that would greatly benefit from more stringent advertising restriction policies to combat social inequalities^(35)^.

When we broadened our search to the field of nutrition, we identified some initial studies that also used AI-based algorithms for food and non-food image classification through photos taken by the individuals themselves to facilitate the identification of food intake and use this information in dietary analysis. The results of these researches using AI-based techniques also proved to be promising^(36,37)^ and illustrate the importance of incorporating new technologies to solve complex public health problems in an autonomous or semi-autonomous way.

Regarding our study’s practical implications, they can be pointed out to researchers, public health policymakers, and regulatory bodies in Brazil and other countries. Although our methodology and results do not yet allow an assessment of the adequacy of advertising in Brazil, they can already help filter food advertising by reducing the number of ads that need to be analysed individually. In future studies, researchers can move forward with identifying non-compliance with advertising regulations. Policymakers of other countries can also use this methodology as inspiration to propose a specific test for the reality of each country and help them in the proposal and approval of more specific policies to promote a healthy food environment.

Researchers can also be inspired by our experience to classify ads by food type and advance in recognising the main patterns of persuasive strategies used in advertising through more effective and efficient models to address these issues. Based on our preliminary observations of the algorithm and data imbalance strategies employed, we believe that certain marketing strategies can be easily identified through automated monitoring. For instance, adhering to the INFORMAS protocol, objective strategies such as the presence of cartoons or company-owned characters, licensed characters, celebrities, renowned sportspersons and sports events can be efficiently detected utilising a robust training database. From our current understanding, it is evident that these specific marketing techniques exhibit distinct features that lend themselves to automated identification. By leveraging a comprehensive and well-curated database, the training process can be optimised to recognise and classify such strategies accurately. These new models should be implemented and tested similarly to this study, using a training dataset, a validation dataset and a testing dataset. To improve the accuracy and robustness of the models, researchers could consider incorporating additional features such as nutritional content, product placement, and endorsement by celebrities or influencers. Moreover, future studies could: *(i)* classify and differentiate between healthy and unhealthy food products by applying established nutritional profile criteria, such as those provided by the WHO^(38)^, the Pan American Health Organization (PAHO)^(39)^ and/or using the NOVA food classification^(40)^; *(ii)* explore the feasibility of automatic monitoring of food advertising on online platforms such as social media, YouTube and streaming services; *(iii)* incorporate advanced techniques such as natural language processing and sentiment analysis to broaden knowledge of the impact of food marketing; and *(iv)* train algorithms to recognise and categorise specific food brands in advertisements. This latter point becomes particularly relevant as food advertising regulations evolve, with emerging policies designed to restrict the promotion of unhealthy foods and their nutritional content. As a result, the industry may increasingly prioritise branded marketing over food product promotion.

By combining our current findings with future research, the application of ML techniques for automatically monitoring food ads can support researchers in extending systematic monitoring and facilitating comparability across studies. A database with different types of food ads shown on TV can be used in comparative studies between countries to identify common marketing patterns used by food companies in different cultural and regulatory contexts. In the medium and long term, the practical implications of advertising monitoring studies, especially those with a large volume of data, could contribute to a healthier food environment and result in lower rates of obesity by minimising individual exposure to food advertising.

Despite the promising results of our study on food advertising monitoring, there are still areas where further progress is needed. Consequently, our future endeavours will focus on the following steps: *(i)* implement the tested algorithm in a new round of data collection to propose an enhanced monitoring approach; *(ii)* expand our existing database, particularly in terms of food ads, thereby enabling greater automation of the monitoring process; *(iii)* apply the algorithm to video datasets from other countries with similar food marketing contexts to evaluate its effectiveness, identify potential necessary adaptations, and facilitate cross-country comparisons and method harmonisation for automated advertising monitoring in diverse settings; and *(iv)* develop user-friendly software interface for the algorithm, streamlining its usability and ensuring efficient operation across a range of machine configurations adaptable to researchers’ requirements.

Collaboration with researchers from various countries is instrumental in expanding the database and developing technologies that can accelerate the automatic tracking of ads across different media platforms and geographical locations. By combining efforts, we can advance the field and contribute to developing effective tools for comprehensive and efficient food advertising monitoring.
